# Do the oral *Staphylococcus aureus* strains from denture wearers have a greater pathogenicity potential?

**DOI:** 10.1080/20002297.2018.1536193

**Published:** 2018-10-23

**Authors:** Katarzyna Garbacz, Tomasz Jarzembowski, Ewa Kwapisz, Agnieszka Daca, Jacek Witkowski

**Affiliations:** a Department of Oral Microbiology, Faculty of Medicine, Medical University of Gdańsk, Gdańsk, Poland; b Department of Medical Microbiology, Faculty of Medicine, Medical University of Gdańsk, Gdańsk, Poland; c Department of Pathology and Experimental Rheumatology, Faculty of Medicine, Medical University of Gdańsk, Gdańsk, Poland; d Department of Pathophysiology, Faculty of Medicine, Medical University of Gdańsk, Gdańsk, Poland

**Keywords:** Dental prosthesis, *Staphylococcus aureus*, phagocytosis, monocytes, stomatitis

## Abstract

We used flow cytometry to compare the phagocytic activity of monocytes against *Staphylococcus aureus* strains (both biofilm and planktonic cells) isolated from denture wearers and non-wearers. Staphylococcal strains were cultured in Brain Heart Infusion broth in both planktonic and biofilm form and were stained with a fluorescent reporter (propidium iodide) and incubated with monocytes. The fluorescence of the monocytes containing phagocytized bacteria was determined by flow cytometry and normalized to that of the bacterial strains used in the experiment. Staphylococcal strains from denture wearers caused greater activation of monocytes but were less prone to phagocytosis. The percentage of monocytes containing bacterial cells after exposition to staphylococcal strains varied from 2.7% to 81.4% for planktonic cells. For biofilm-released cells, this value ranged from 0.6% to 36.2%. The effectiveness of phagocytosis, estimated based on an increase in monocyte fluorescence, amounted to 32.4 and 71 FL2 units for the biofilm and planktonic cells, respectively. The lesser efficiency of phagocytosis against biofilm *S. aureus* in denture wearers suggests that they might have been colonized with the strains which were less prone to eradication than those from non-wearers.

Phagocytosis is the main mechanism for protection against oral pathogens. Inflammatory monocytes play a key role in innate immune defence against microbial infection and also contribute to adaptive immune response. The pleomorphic and pleiotropic population of circulating mononuclear cells participates in antimicrobial defence, supplying tissues with precursors of macrophages and dendric cells. However, in case of infection, the homoeostatic pathway of differentiation is temporarily rearranged, and bone marrow and blood monocytes differentiate into an array of effector cells with distinct antimicrobial properties [1]. A main function of monocytes is ingestion and destruction of pathogens. Unlike neutrophils, monocytes survive longer at an acute inflammation site and due to their diverse capacity to kill microbes undisputedly constitute an essential component of the innate immune system [2]. Noticeably, our previous study demonstrated that monocytes respond differently to commensal and invasive strains [3].

The mouth’s properties as a microbial habitat are dynamic and change over the lifetime. Previous studies documented direct age-related changes in the oral microbiota, such as an increase in the proportion and isolation frequency of some bacterial species, primarily *Staphylococcus aureus* [4]. *S. aureus* may cause a plethora of infections, among them oral diseases, such as angular cheilitis, mucositis, periodontitis and dental implant-associated infections [5]. However, the biology of oral *S. aureus*, and especially the changes they undergo under specific intraoral conditions in elderly denture wearers, is still not fully understood. The aim of this study was to compare by flow cytometry the phagocytic activity of monocytes against *S. aureus* strains (both biofilm and planktonic cells) isolated from denture wearers and non-wearers.

Between March 2016 and April 2017, a total of 38 oral *S. aureus* strains were collected during routine laboratory procedures at the Department of Oral Microbiology, Medical University of Gdańsk. Written informed consent was sought from all the study participants, and the protocol of the study was approved by the Local Independent Committee for Ethics in Scientific Research at the Medical University of Gdańsk. Each strain originated from a different patient (*n* = 38). The study included solely adult patients with the signs of oral infection, from whom no other pathogenic bacteria or fungi were isolated except *S. aureus*. Individuals with periodontal disease and/or dental implants were excluded from the study. Twenty staphylococcal strains were isolated from the oral cavity of denture wearers (Group 1), among them 15 from patients with denture stomatitis (DS), and another 18 strains were collected from the non-wearers (Group 2). Median age of denture wearers was 67.8 years (range 46–82 years) and patients in group II was 51.8 years (range 24–69 years).

The samples were subcultured onto Columbia blood agar and Chapman agar and incubated at 37°C for 24–48 h. Identification of *S. aureus* was based on colony morphology, Gram staining, agglutination with the latex test Pastorex Staph Plus (Bio-Rad, France) and testing with the API ID32 Staph system (bioMerieux, France). The strains were ultimately identified as *S. aureus* based on polymerase chain reaction amplification of the species-specific *nuc* gene [6]. After the identification, the strains were stored at −80°C in Tryptic Soy Bullion with 15% glycerol.

Phagocytosis assay was conducted on the THP-1 cell line (ATCC). The cells were cultured in RPMI-1640 medium supplemented with 2 mM L-glutamine, 100 U/ml penicillin, 100 µg/ml streptomycin and 10% (vol/vol) heat-inactivated foetal bovine serum. All reagents were purchased from Sigma-Aldrich (Germany).

Each phagocytosis assay included staphylococcal strains cultured in the planktonic and biofilm form. The overnight cultures in Brain Heart Infusion (BHI) broth were inoculated into fresh BHI broth and grown to a mid-log phase in a planktonic form (18 h at 37°C). Bacterial biofilms were prepared in six-well tissue culture plates (TPP Techno Plastic Products, Switzerland). The wells were filled with 2 ml of BHI broth, inoculated with the overnight culture and incubated at 37°C for 24 h. Then, 1 ml of the culture was replaced with the fresh broth and incubated for another 48 h. Finally, the broth was removed, and the wells were washed with saline. The biofilm structure was released into fresh saline by sonication and stained with propidium iodide (PI). Also, the planktonic cells were washed with saline, permeabilized by sonication on ice and stained with PI at room temperature. After staining, the bacteria were centrifuged to remove the excess of reagents and resuspended in saline.

Each phagocytic mixture contained 0.4 ml of the stained staphylococci (approximately 8 × 10^7^ cells) and 0.4 ml of monocytes (2 × 10^4^ cells) suspensions. The mixture was incubated at 37°C for 45 min, and then, the fluorescence of the THP-1 cells was determined with a FACScan flow cytometer (Becton-Dickinson, Franklin Lakes, USA). The fluorescence of monocytes resulting from phagocytosis of the bacteria (FL2) was normalized to the fluorescence of the bacterial strain used in the experiment. A PI-stained suspension of monocytes without bacteria was used as control: the range of FL2 for such a suspension was assumed to be negative.

The results were subjected to the analysis of variance (ANOVA) with Statistica 10 software (StatSoft, USA). The normality of distribution was verified (*χ*^2^ test) and the data were normalized by Box–Cox transformation.

The percentage of monocytes containing bacterial cells after exposition to studied strains varied from 2.7% to 81.4% for planktonic cells. For biofilm-released cells, this value was lower and ranged from 0.6% to 36.2%. The effectiveness of phagocytosis, estimated based on an increase in monocyte fluorescence, amounted to 32.4 and 71 FL2 units for the biofilm and planktonic cells, respectively (Figure 1).

**Figure 1.**
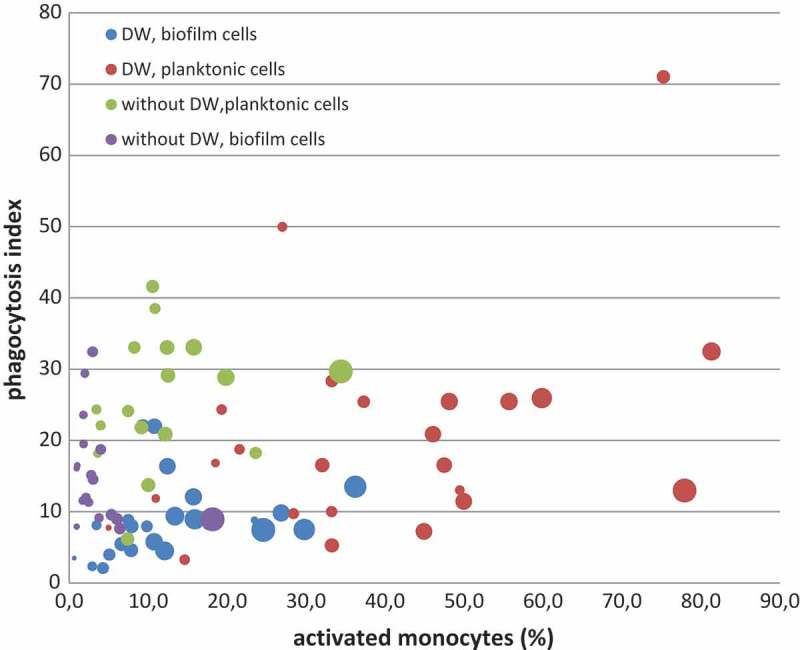
Diversity of monocyte response to *S. aureus* strains from Group 1 and Group 2. One spot represents the result (median) for phagocytosis of a single strain, the size of the spot corresponds to the size of the sample (number of measured monocytes).

Staphylococcal strains from denture wearers (Group 1) caused greater activation of monocytes than from non-wearers (Group 2) but were less prone to phagocytosis. Although this referred to both planktonic and biofilm forms, the biofilm bacteria showed greater resistance to phagocytosis than the planktonic cells (Figure 2).

**Figure 2.**
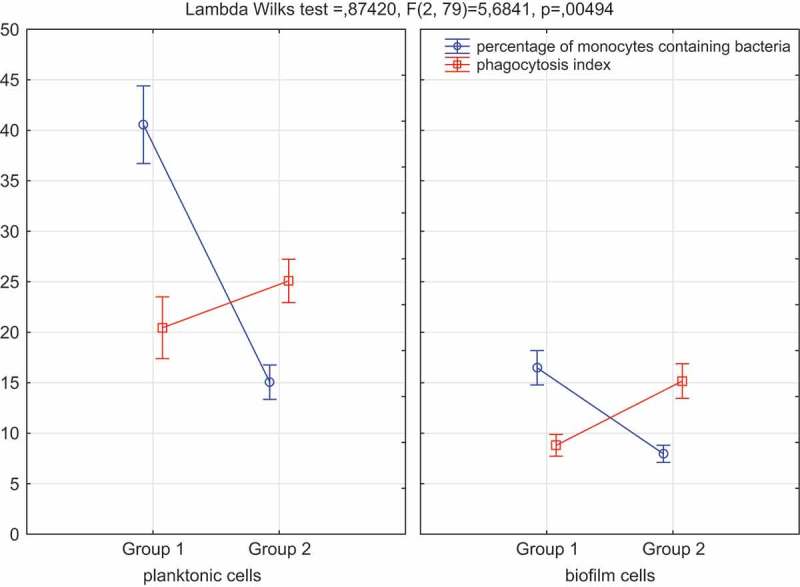
ANOVA comparison of phagocytosis of *S. aureus* and monocyte activation.

Although dentures are used by a large proportion of older persons [7], the changes in the oral microbiota of denture wearers are still not completely understood. Even less is known about adaptive changes of bacteria colonizing oral cavities of denture wearers. Distinguishing between the changes related directly to the denture use and those resulting from ageing can be particularly challenging. Not surprisingly, the mean age of denture wearers participating in our study was significantly higher than the age of non-wearers. Oral microbiota may be modulated by an array of age-related factors, both directly (e.g. due to a decrease in salivary and serum antibody levels) and indirectly (e.g. due to medication use or changes in diet). As a result, in subjects older than 70 years, some bacteria, among them *S. aureus*, are isolated from saliva more often than in younger people [8,9].

For many years, researchers believed that colonization of the oral cavity with *S. aureus* does not exert a profound effect on the mouth’s condition. However, the results of studies conducted in the recent decade suggest that *S. aureus* colonizes the oral cavity more frequently than expected (more often than the nasal vestibule), which may be associated with various systemic and oral infections [10]. *S. aureus* is more often the component of the oral microflora in denture wearers than in non-wearers [11]. Furthermore, recent evidence suggests that *S. aureus* strains are isolated equally frequently from older persons presenting with DS as from asymptomatic denture wearers [11]. This sheds new light on our findings, as we isolated staphylococci from both the symptomatic and asymptomatic denture wearers. However, these isolates were less susceptible to phagocytosis by monocytes than the strains from the non-wearers. This implies that adaptation of *S. aureus* to specific conditions of the oral cavity in denture wearers may lead to a preferential selection of some strains and/or changes in their properties.

*S. aureus* can adhere to a wide variety of oral surfaces, including dental prostheses [12]. As a non-shedding oral surface, the denture is particularly easily colonized with staphylococcal biofilm [7]. Our findings support the notion that phagocytosis of staphylococcal biofilm cells from denture wearers is less effective, despite a stronger monocyte response than in non-wearers. This implies that despite triggering a stronger monocyte response, biofilm staphylococci from denture wearers are phagocytized less effectively than those from the non-wearers. Many previous studies showed that recruitment of monocytes is essential for effective control and clearance of bacterial infections. In line with those findings, the stronger monocyte response may be considered as evidence for greater virulence of staphylococcal isolates from denture wearers. Due to lower efficiency of phagocytosis, such strains might be less prone to eradication, which provides an explanation for higher rates of staphylococcal colonization/infection reported in elderly patients. According to Smith et al. [5], denture-colonizing staphylococci may constitute a potential reservoir for transmission to other body sites, e.g. to the respiratory tract. Indeed, published evidence suggests that biofilm fragments from the denture surface may be delivered to the lungs, which results in systemic infections, such as aspiration pneumonia [10].

In summary, our findings suggest that the oral cavity in denture wearers, even in persons without DS, may be colonized by *S. aureus* strains which are probably less prone to eradication than those from non-wearers. Presumably, in the case of older people, who are generally more susceptible to cardiovascular and respiratory diseases, this colonization may constitute one of many risk factors for staphylococcal infections.
